# Method to improve critical current density measurement of superconducting materials^✰^

**DOI:** 10.1016/j.mex.2023.102087

**Published:** 2023-03-02

**Authors:** Yunkai Shao, Longxiang Liu, Sansheng Wang

**Affiliations:** School of Physics, Beihang University, Beijing 102206, China

**Keywords:** Double-sided measurement method based on the mutual induction, Superconducting film, Critical current density, Third harmonic voltage, Signal-to-noise ratio

## Abstract

The critical current density J_c_ is an important indicator of superconducting materials in practical applications. And the third harmonic voltage method is a simple, efficient and damage-free method for measuring J_c_ of high-temperature superconducting films. In order to more effectively measure the J_c_ of high-temperature superconducting REBa_2_Cu_3_O_7-δ_ films and improve the signal-to-noise ratio based on the third harmonic voltage method, a double-sided measurement method was hereby proposed. By integrating the electromagnetic shielding effect and the 2-coil measurement system, the Meissner effect was used to reduce the harmonic voltage noise in the pickup coil. Comparing the double-sided measurement system using the new method to the 2-coil measurement system, the signal-to-noise ratio increases by 10 dB to 20 dB in the case of a J_c_ measured in the frequency range from 100 Hz to 1000 Hz. The bullet points of the method can be listed as:

• The noise in the pickup coil is reduced and the signal-to-noise ratio is improved in the frequency range from 100 Hz to 1000 Hz by using the Meissner effect.
• The measurement bandwidth is increased, making it possible to conduct low-frequency measurement in the future by the double-sided measurement method.
• The method provides an improvement direction for superconducting thin films with a high critical current density in the future.

## Graphical abstract

**Figure:**
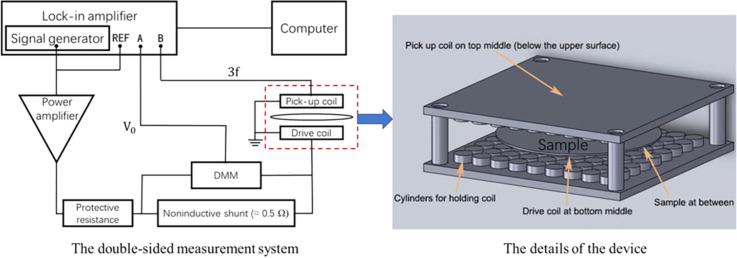

Specifications table

**Table:** 

Subject area:	Materials Science
More specific subject area:	high-temperature superconducting REBa_2_Cu_3_O_7-δ_ films
Name of your method:	Double-sided measurement method based on the mutual induction
Name and reference of original method:	Not applicable
Resource availability:	Not applicable

## Background

The critical current density J_c_ is one of the important parameters to evaluate the application value of superconducting materials, but it always remains challenging to accurately measure the J_c_ of superconducting films. J. H. Classen [1] proposed two critical current measurement methods in 1991, the self-induction method and the mutual induction method. Some researchers [2], [3], [4], [5], [6] subsequently studied superconducting films by the self-induction method using a single coil that acts as both a drive coil and a pickup coil on one side of the superconducting films. Meanwhile, the models of various measurement systems were also proposed after continuous improvement, which roughly fall into three different types: (1) the double-film measurement system [7]; (2) the variable-RL-cancel circuit measurement system [6]; and (3) the 2-coil measurement system. The double-film measurement system performs excellently in reducing the harmonic noise generated by the signal generator and the power amplifier using a cancel coil furnished with the superconducting film with a high J_c_ as the control group, which, however, requires an extra superconducting film. As pointed out [8], a cancel coil without a superconducting film can also be used for effectively reducing the noise. Although by using variable resistance and variable inductance coils to simulate the self-inductance and resistance of the sample, the variable-RL-cancel circuit measurement system also performs well in noise reducing, its circuit is still too complicated. The 2-coil measurement system [7] fails to reduce the noise generated by inductive reactance, though it has a rather simple circuit that uses a pick-up coil and a drive coil to eliminate the effect of resistance on harmonic noise.

The mutual induction method, known as the sandwich model, was firstly proposed by P. Scharnhorst [9] in 1970 for measuring the critical current density of low-temperature superconductors. The superconducting film is sandwiched between the drive coil and the pick-up coil, and its surface information is obtained using the electromagnetic shielding effect. H. Yamada et al. [10] studied whether an iron core could be used to enhance the electromagnetic field and reduce the current in the circuit using the mutual induction method. However, there is always noise in the circuit, which will inevitably affect the measurement results, thereby making noise reduction in critical current density measurement of superconducting materials still a key problem. In this case, the mutual induction method was improved and a new measurement device with a simple circuit was correspondingly designed to reduce the noise as efficiently as possible.

## Method details

Required equipment

• Lock-in amplifier
• Signal generator
• Power amplifier
• Protective resistance
• Noninductive shunt
• Drive coil
• Pick-up coil
• Computer
• Digital multipurpose meter
• Refrigerator
• Liquid nitrogen

Preparation of Samples

• High-temperature superconducting films (with a diameter of about 2 inches and a thickness of about 200 nm)
• Substrates (0.5 mm LaAlO_3_)

Procedure

**Fig. 1:**
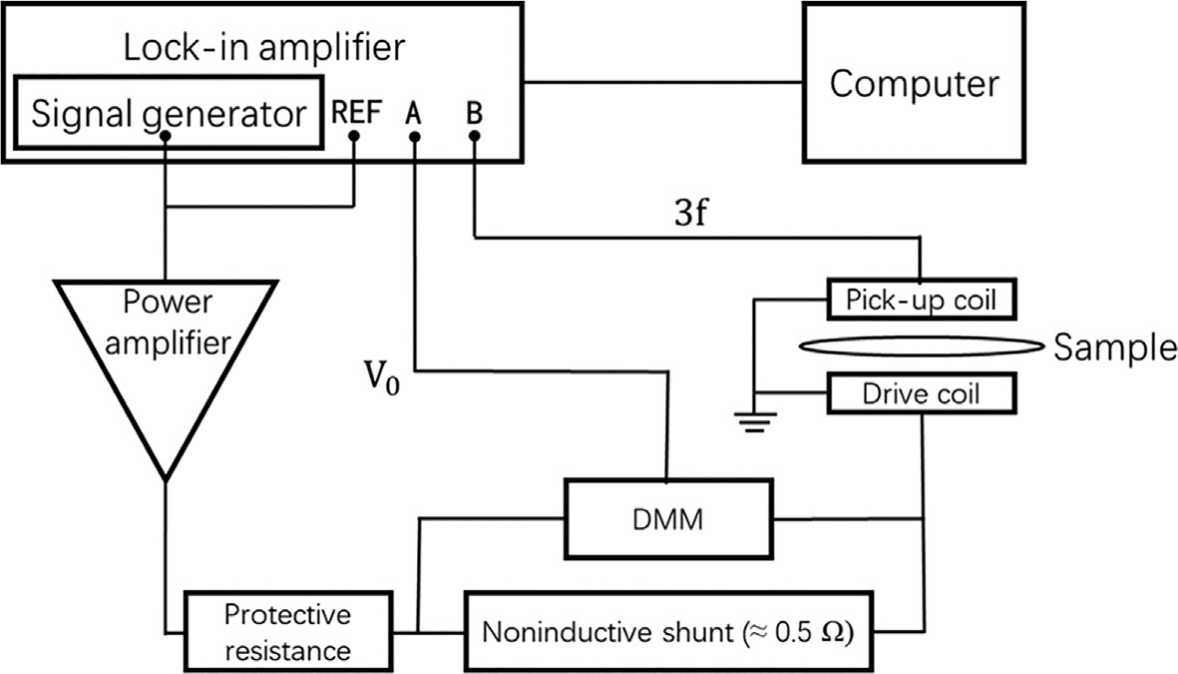
Double-sided measurement system[11].

1. Set up the Double-sided measurement system according to the circuit diagram, as shown in Fig. 1. If you want to compare the double-sided measurement system and the typical 2-coil measurement system which is shown in Fig. 2 in terms of noise reduction, all you need to do is place the drive coil and the pick-up coil on the same side of the sample, which is a simple 2-coil measurement system.

**Fig. 2:**
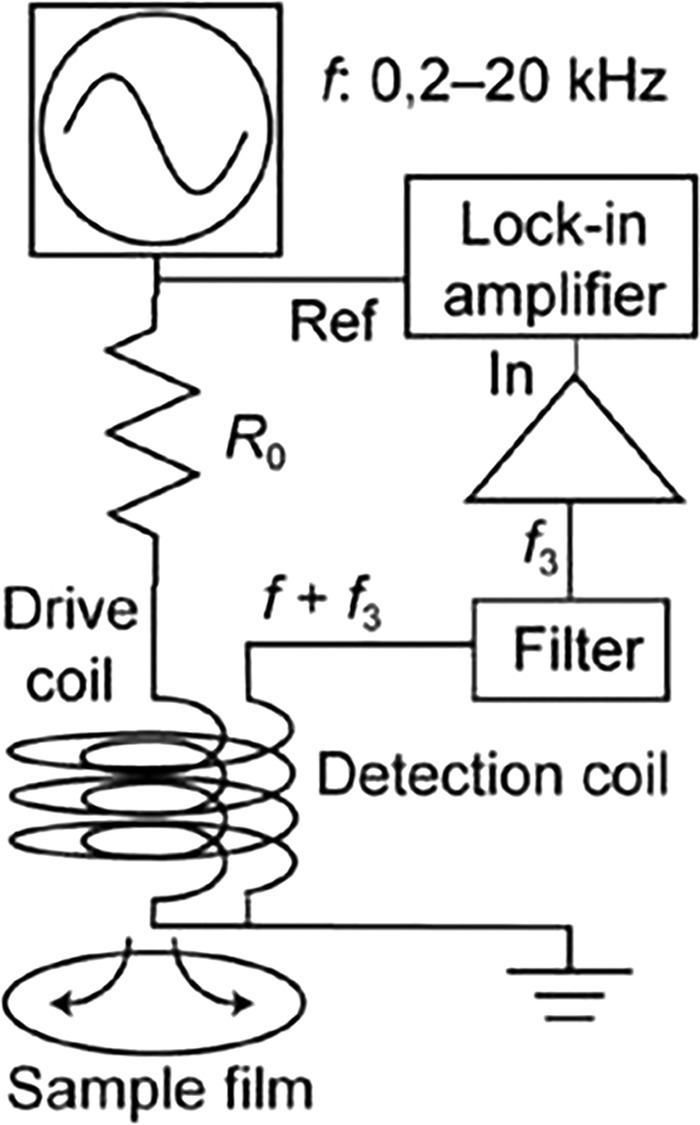
The typical 2-coil measurement system[7].
2. Put the drive coil, the pick-up coil and the sample according to Fig. 3. The superconducting film is sandwiched between these two coils at a distance of 0.4 mm from the upper and the lower coils. The cylinder is used to fix the coil, and only the center cylinder has a coil while measuring.

**Fig. 3:**
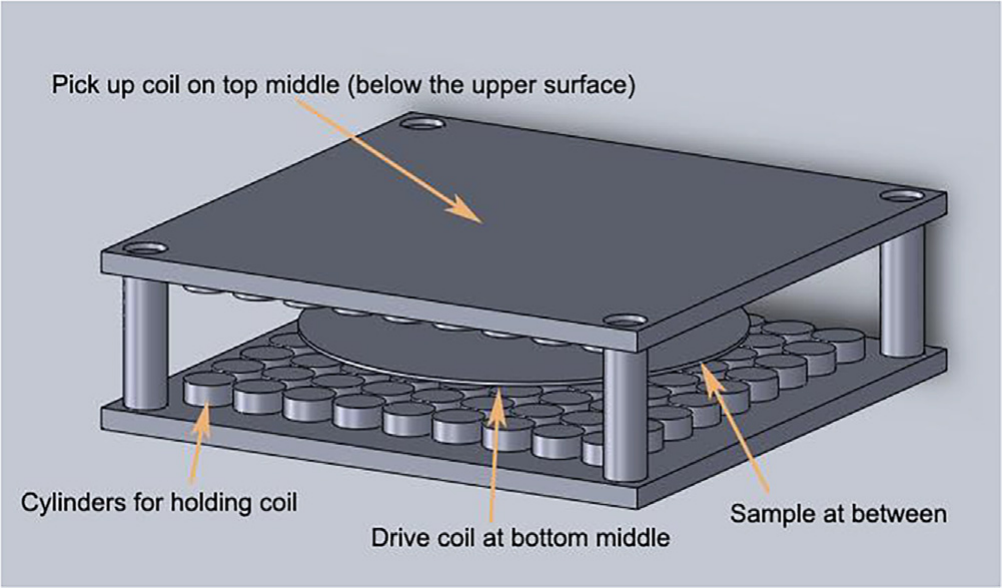
Coil and sample diagram [11].
3. Make the temperature of the sample reach the phase transition point. The temperature of the sample here is 78 K. Refrigerator can be used to cool the whole device shown in Fig. 3, and the sample can also be immersed in liquid nitrogen. After the sample is in the full superconducting state, the next step can be carried out.
4. (Optional - calibration) This step should be performed for the re-calibration of the device after structural changes. In the previous step, a sample with a known critical current density J_c_ (such as the adoption of a four-probe method) is used for the subsequent steps.
5. Select the appropriate measuring range according to the actual situation. In the present experiment, the output frequency f of the signal generator ranges from 200 Hz to 2000 Hz, and the DMM voltage varies from 10^−5^ *V* to 10^−1^ *V*. For the sample with good performance or long test time, the researchers may need to cool down the protection resistance and shunt resistance R_0_. According to the reference signal, the lock-in amplifier separates the signal of Channel A and Channel B from the same frequency signal V_1_ and the third harmonic signal V_3_.
6. Use $V_{3}/\sqrt{2}f$ as ordinate and $I_{0}=V_{1}/\sqrt{2}R_{0}$ as abscissa for drawing, as shown in Fig. 4.

**Fig. 4:**
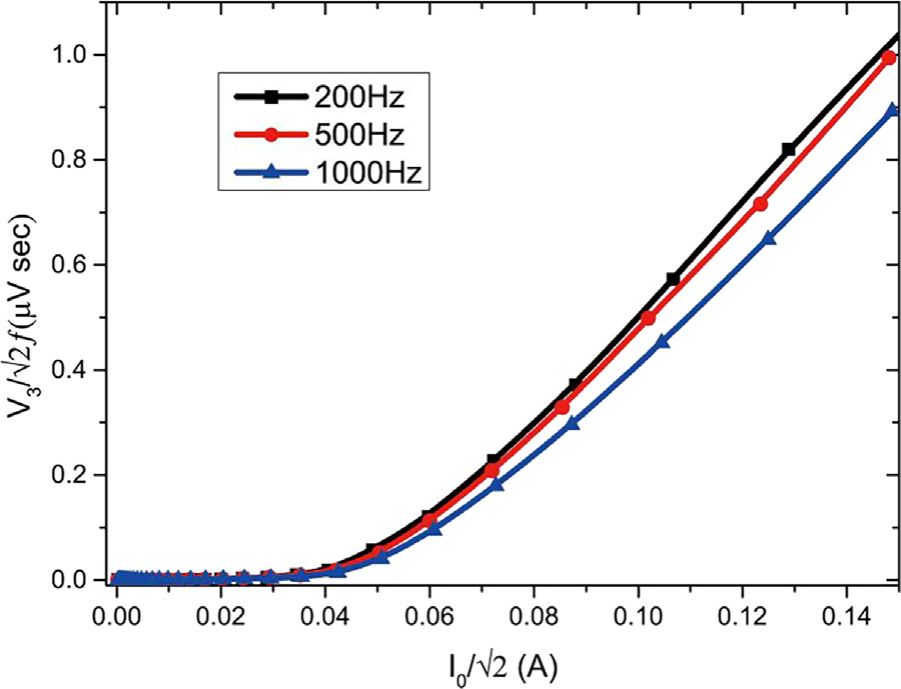
Third harmonic diagram [11].
7. Select an appropriate standard as the phase transition point. In Fig. 4, 0.2*μVsec* is used, and I_c0_ corresponding to 0.2*μVsec* is obtained. The critical current density is calculated as $J_{c}=\;I_{c0}\times k$. (Follow the same steps to get $k=\;J_{c}^{*}\;/\;I_{c0}^{*}$ during the calibration).

Note: The geometric size of the samples we used are 200 nm thick and 2 inches in diameter. According to the theory mentioned in Reference 1, if the diameter of the sample is greater than twice the outer diameter of the coils, then the edge effect is negligible. We therefore recommend that readers use a sample larger than twice the outer diameter of the coils. In terms of thickness, please do not use samples with thickness below the penetration depth of London. Our sample transition temperature is about 90 K, so we immersed the sample in liquid nitrogen and did not use a refrigerator. If you want to use liquid nitrogen instead of a refrigerator, liquid nitrogen should be kept at a relatively stable temperature at the beginning of measurement to avoid the measurement effect caused by liquid nitrogen boiling.

## Method validation

Two superconducting film samples labeled ‘**a**’ and ‘**b**’ were prepared, and a set of custom-made coils were bought. Superconducting film **a** is two inches in diameter and 200 nm in thickness, YBa_2_Cu_3_O_7-δ_, J_c_ ≈1.6 MA/cm^2^; the size of superconducting film **b** is the same to **a**, DyBa_2_Cu_3_O_7-δ_, J_c_ ≈2.4 MA/cm^2^. The J_c_ of the samples were measured using THEVA equipment. Both superconducting films **a** and **b** used 0.5 mm LaAlO_3_ as the substrate. The pickup coil and the drive coil are the same, the inner diameter is 1.5 mm, the outer diameter is 6 mm, and the height is 2.5 mm. The number of turns of the copper coil is 420, and the wire diameter is 0.1 mm.

Table 1 shows the signal-to-noise ratio of the double-sided measurement device and the 2-coil measurement device for measuring the J_c_ of the superconducting films **a** and **b**. It reveals that the signal-to-noise ratio of the double-sided measurement system is higher than that of the 2-coil measurement system at the same frequency, with a higher frequency indicating a higher signal-to-noise ratio. The 2-coil measurement system performs poorly in measuring the J_c_ of the film **a** with a low critical current density, which, however, could be effectively measured by the double-sided measurement system.

**Table 1:** The signal-to-noise ratio of the two devices using different methods [11].

		frequency	threshold criterion (μV*sec)	threshold current I_c0 (_A)	noise (μV*sec)	signal-to-noise ratio (dB) 20Log (V_S_/V_N)_
film **a**	2-coil measurement system	200Hz	0.2	0.15	0.2142	Completely distorted
		500Hz	0.2	0.15	0.3889	Completely distorted
		1000Hz	0.2	0.15	0.5459	Completely distorted
	Double-sided measurement system	200Hz	0.2	0.15	0.037	12.8797
		500Hz	0.2	0.15	0.0134	22.8761
		1000Hz	0.2	0.15	0.0136	22.7381
film **b**	2-coil measurement system	200Hz	0.2	0.04	0.0138	22.5986
		500Hz	0.2	0.04	0.01562	15.0609
		1000Hz	0.2	0.04	0.03002	21.4394
	Double-sided measurement system	200Hz	0.2	0.04	0.01257	23.4685
		500Hz	0.2	0.04	0.00333	35.4372
		1000Hz	0.2	0.04	0.00216	39.2433

## Conclusion

The critical current density J_c_ of high-temperature superconducting films measured using the third harmonic voltage method is greatly affected by noise. In this case, a new measurement method, known as the double-sided measurement method, was hereby proposed based on the 2-coil measurement system, which greatly reduces the noise in the pick-up coil and improves the measurement accuracy by means of the Meissner effect. Given that more information about the superconducting film can be obtained by low-frequency measurement, the method can be used as a template for the low-frequency measurement system. The method validation proves that the double-sided measurement method is accurate and simple, and is endowed with great potential in practical applications. Additionally, this new method also provides an improvement direction for superconducting thin films with a high critical current density in the future.

## CRediT authorship contribution statement

**Yunkai Shao:** Validation, Visualization, Writing – original draft. **Longxiang Liu:** Validation, Formal analysis, Writing – review & editing. **Sansheng Wang:** Conceptualization, Methodology, Project administration.

## Declaration of Competing Interest

The authors declare that they have no known competing financial interests or personal relationships that could have appeared to influence the work reported in this paper.

## Data Availability

The data that has been used is confidential.
